# Study on Anti-Aging Performance Enhancement of Polymer Modified Asphalt with High Linear SBS Content

**DOI:** 10.3390/polym15020256

**Published:** 2023-01-04

**Authors:** Daqian Han, Guosheng Hu, Jingting Zhang

**Affiliations:** School of Materials Science and Engineering, North University of China, Taiyuan 030051, China

**Keywords:** modified asphalt with high linear SBS content, nanoparticles, PPA, anti-aging properties, fatigue properties, infrared spectroscopy test

## Abstract

Modified asphalt with high content SBS is widely used in asphalt pavement due to its excellent high and low temperature performance. However, its anti-aging performance is insufficient. In order to improve the anti-aging performance of SBS modified asphalt, nano-ZnO, nano-TiO_2_, nano-SiO_2_ and polyphosphoric acid (PPA) were added to high content (6.5 wt%) linear SBS modified asphalt as anti-aging agents in this study. Moreover, Dynamic Shear Rheometer (DSR), Fluorescence Microscope, and Fourier Transform Infrared Spectroscopy were employed to reveal the mechanism, through the investigation of the rheological and microscopic properties of modified asphalt before and after aging. The results showed that the influence of nanoparticles on the rutting resistance and fatigue resistance of high content SBS modified asphalt is weak, mainly because there is only weak physical interaction between nanoparticles and the SBS modifier, but no obvious chemical reaction. The significant cross-networking structure of high content SBS modified asphalt even has an adverse effect on the anti-aging performance of nano-modifiers. However, PPA obviously makes the cross-linked network structure of SBS modified asphalt more compact, and significantly improves the performance after short-term aging and long-term aging, mainly due to the chemical reaction between PPA and the active groups in SBS modified asphalt.

## 1. Introduction

Polymer modified asphalt with high content SBS (HCPMA) is a promising asphalt binder in the open-graded friction course (OGFC) pavement due to excellent high temperature shear resistance and adhesion characteristics [1,2,3]. However, the potential weak aging effect of HCPMA hinders the widespread application [4]. Studies have shown that HCPMA has active chemical properties, which are sensitive to oxygen, and could be degraded easily in high-temperature environments. Moreover, light components volatilize, and the active groups in asphalt react with oxygen during aging, leading to the content increase of carbonyl and sulfoxide functional groups. The average molecular weight of asphalt after aging increases due to intermolecular condensation and polycondensation, the content of heavy components increases, and the elasticity of asphalt decreases. Asphalt becomes brittle and hard, resulting in low-temperature cracking of the pavement [5,6]. Recent studies also showed that after long-term aging, the asphalts were sharply deteriorated and the stripping potential per unit area became overly high, resulting in a remarkable reduction in moisture resistance [7]. As a result, its overall pavement life is only 60~70% of the ordinary dense-graded pavements [8]. Therefore, it is very important to further improve the anti-aging performance of HCPMA.

In recent years, researchers have devoted themselves to improving the anti-aging performance of modified asphalt by adding anti-aging agents [9,10,11]. Nano materials form stable structures with other atoms due to interfacial effects. Liu selected modified nano-TiO_2_ as an anti-aging agent to study the influence of the sunlight and oxygen aging of modified asphalt [12]. The study found that the (colloidal index) of modified nano-TiO_2_/modified asphalt decreased after UV aging. In addition, compared with the unmodified nano-TiO_2_, the modified nano-TiO_2_ had a superior anti-aging effect. Zhang found that nano-ZnO could significantly promote the anti-ultraviolet light aging ability of modified asphalt, but the improvement effect of its thermal oxygen aging resistance was slight [13]. Eillie’s research presented how nano-SiO_2_ effectively enhanced storage modulus capability and elastic behavior, thereby enhancing rutting resistance [14]. Meanwhile, the variation in rheological properties and the infrared spectrum aging index of aged nano-SiO_2_/modified asphalt became slow. Furthermore, many researchers have discussed the anti-aging mechanism of nanoparticles for SBS modified asphalt. Zhang considered how nanoparticles with high surface energy and large active specific surface area have a strong bonding ability to both asphalt and polymer particles. The addition of nanoparticles can form an oriented film between the polymer and asphalt, thus reducing the interfacial tension between the polymer and asphalt and improving the dispersion of polymer particles [15]. However, Guo pointed out that the influence of nano materials on the properties of SBS modified asphalt is not only related to physical changes, but also related to chemical reactions [16]. Besides, in the past 30 years, PPA has attracted much attention from researchers due to its low price and excellent anti-aging performance [17,18,19]. Ramasamy B.S. has studied the rheological and aging properties of unmodified and modified asphalt [20]. The results showed that PPA enhanced the mechanical and anti-aging properties of modified asphalt. However, PPA was unfriendly to low-temperature and the fatigue properties of modified asphalt. As for the mechanism of the influence of PPA on the performance of SBS modified asphalt, most researchers believed that it is a chemical reaction between PPA and active groups (such as sulfoxide group) in SBS modified asphalt [21,22].

Based on the above analysis, nanoparticles and PPA can enhance the anti-aging performance of low content linear SBS modified asphalt. However, the influence and mechanism of anti-aging agents on the performance of high content linear SBS modified asphalt is almost blank. Therefore, in this study, nano-ZnO, nano-TiO_2_, nano-SiO_2_ and PPA with 115% orthophosphoric acid were selected as anti-aging modifiers to prepare modified asphalt with a high content of linear SBS. Then, the anti-aging properties were comprehensively evaluated by DSR rheometer, fluorescence microscope and infrared spectrometer. The influencing mechanism of nanoparticles and PPA on the anti-aging properties of high content SBS modified asphalt was revealed.

## 2. Materials and Methods

### 2.1. Experiment Materials

Zhonghai 70# (PG64-16) acted as the base asphalt. Linear SBS 791-H was produced by Sinopec. The solubilizer was selected from rubber oil with 3.0 wt% of base binder produced in Iran. The sulfur was employed as a stabilizing agent with 0.1 wt% of Zhonghai 70#. The content of SBS was 6.5 wt% of Zhonghai 70#. Nano-ZnO, nano-TiO_2_ and nano-SiO_2_ were chemically surface-modified to reduce the specific surface area and promote dispersion. PPA was used with a mass fraction of H_3_PO_4_ of 115%.

There were main three steps involved in the preparation of composite modified asphalt with a high content of linear SBS (6.5 wt%). Firstly, the SBS and rubber oil were blended to Zhonghai 70# and sheared for 35 min at the rate of 5000 r·min^−1^ and 185 °C with a high-speed disperser. Secondly, weighed inorganic nanoparticles, such as nano-ZnO or PPA, were added to the asphalt and sheared continuously for 30 min. The content of all anti-aging agents in this paper was 2.0% of the total amount of base asphalt. Thirdly, the sulfur was added to the mixture and stirred for 120 min at the rate of 2000 r·min^−1^. The final samples were represented by SBS, ZnO/SBS, TiO_2_/SBS, SiO_2_/SBS or PPA/SBS, respectively.

### 2.2. Binder Test Descriptions

According to the AASHTO standard, the short-term aging (rotating film oven aging (RTOT)) and long-term aging (pressure aging (PAV)) were carried out for the original asphalt in this paper, respectively. Then, the rheological properties of the asphalt were analyzed by DSR. The anti-aging mechanism of the high content SBS polymer composite modified asphalt was expounded from a microscopic perspective through fluorescence microscopic images and Fourier transform infrared spectroscopy.

#### 2.2.1. Dynamic Shear Rheological Test

The rheological properties of the modified asphalt were investigated by the HR10 DSR rheometer (TA Instruments, New Castle DE, United States). The dynamic shear modulus G* and phase angle δ are taken as the basic parameters to characterize the rheological properties of asphalt. The G*/sinδ and G*·sinδ characterize the rutting resistance and fatigue resistance of asphalt, respectively. At the same time, the high temperature PG of the original asphalt and short-term aging asphalt were based on the rutting factor.

The high-temperature rutting resistance of modified asphalt was characterized by the multiple stress creep recovery (MSCR) test. The method was performed continuously at 0.1 kPa and 3.2 kPa, respectively. Every pressure condition was carried out for ten cycles, and each process was 10 s. Deformation recovery rate (R) and non-recoverable compliance (J_nr_) are the main parameters. The test temperature was 82 °C, 88 °C and 94 °C.

According to the AASHTO standard, the linear amplitude sweep (LAS) test was conducted by DSR. The test temperature was 25 °C.

#### 2.2.2. Fluorescence Microscopy Image Acquisition

Fluorescence microscopy can obtain reproducible microstructure images without destroying the internal structure of modified asphalt. The different preparation methods can significantly affect its microstructure under fluorescence microscopy. The molding method and steps of the observation sample in this paper are as follows. Firstly, place a metal container with a diameter of 5~10 mm and a height of 15~20 mm on a horizontal table top. Then, pour different SBS composite modified asphalt samples evenly into metal containers and store them at room temperature for 0.5 h. Finally, absorb the water on the surface of the asphalt sample with absorbent paper for observation; this prevents the distortion of the observed image of the sample caused by the reflection of water. This method can avoid the interference of the surface and internal morphology of the sample in conventional ways.

#### 2.2.3. Infrared Spectroscopy Test

The infrared spectroscopy test was conducted by Bruker Tensor 27 (Thermo Fisher Scientific, Waltham, United States) infrared spectrometer equipped with the full scattering ATR accessory. The test was scanned 32 times in the wavenumber range of 4000 cm^−1^~400 cm^−1^. Each test was repeated 3 times, and the average value was the result. All raw spectra were normalized and baseline corrected.

The semi-quantitative analysis was used to explore the variation in functional groups of the modified asphalt during aging. The carbonyl index *(I_CA_*) and a new characteristic index were selected to analyze the effect of long-term aging (PAV) on the chemical composition of modified asphalt by calculating. The new characteristic index was the SBS degradation index (*I_B/S_*), which uses the polybutadiene peak as the characteristic peak and the polystyrene peak as the reference peak [23]. The study showed that the *I_B/S_* is reasonable for presenting the degradation degree of SBS under an ATR scanning environment [24,25,26]. The different characteristic indices were calculated as follows [23]:

(1) $$I_{CA}=A_{1700}/\sum AR\times 100\%$$

where *A*_1700_ is the infrared absorption peak belonging to the 1700 cm^−1^; the Σ*AR* are the peak areas of the asphalt functional groups in the range of 680~3600 cm^−1^.

(2) $$\;I_{B/S}=A_{965}/A_{700}\times 100\%$$

where the *A_965_* is the absorption peak belonging to the 965 cm^−1^; and where the *A*_700_ is the absorption peak belonging to the 700 cm^−1^.

## 3. Results

### 3.1. Effects of Aging on Rheological Properties of Different High Content SBS Polymer Composite Modified Asphalts

#### 3.1.1. Rheological Analysis

The mechanical and deformation properties of the high content SBS polymer modified asphalt with various anti-aging agents after RTFO aging at 82 °C are shown in Table 1. As we can see from Table 1, the addition of nanoparticles has a slight effect on the failure temperature and rutting factor of modified asphalt, and the PG of each nanocomposite modified asphalt is 82 °C. However, it is worth mentioning that the failure temperature of the PPA/SBS composite modified asphalt is as high as 125.7 °C after adding polyphosphoric acid, which is 46.0% higher than that of SBS modified asphalt. This shows that it possesses extremely outstanding high temperature and aging resistance.

Within the two indicators, phase angle and complex modulus, the complex shear modulus G* characterizes the deformation resistance of asphalt under continuous shear load. The lower the G*, the weaker the deformation resistance. The phase angle δ characterizes the viscoelastic properties of modified asphalt. The δ is 0° for pure elastomers and 90° for pure viscous materials. Table 1 shows that various nanomaterial anti-aging agents, such as nano-ZnO, have no noticeable effect on the G* and δ of SBS modified asphalt. However, adding PPA can significantly reduce the δ and improve G*. Therefore, the elasticity and shear deformation resistance of modified asphalt are enhanced considerably, which means that it will improve the permanent deformation and aging resistance remarkably. The reason is that, PPA can react with small molecules in asphalt and generate a macromolecular structure, which makes the asphalt more elastic [18].

#### 3.1.2. Analysis of MSCR Test Results

(1) R Analysis of deformation recovery

**Figure 1:**
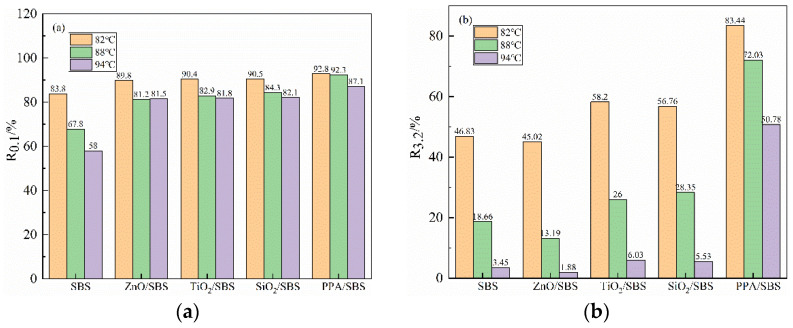
The deformation recovery rate of modified asphalt under different conditions after RTFO aging: (**a**) R_0_._1_; (**b**) R_3_._2_.

To further evaluate the asphalt binder actual rutting resistance level and rheological properties at high-temperature, this study used the R and J_nr_ of the MSCR test. Modified asphalt samples after RTFO aging were selected to test. Furthermore, three different temperature gradients of 82 °C, 88 °C and 94 °C were selected in the test to study the dependence of modified asphalt on temperature. The results are presented in Figure 1.

The higher the R is, the stronger the proportion of the recoverable strain of the asphalt will be, and the smaller the ratio of the residual strain will be. When no anti-aging agent is added at the 0.1% stress level, the R of SBS modified asphalt is relatively low, and the value reduces remarkably with the increase in temperature, as depicted in Figure 1. After adding nanomaterials, such as nano-ZnO or PPA, the R has been significantly increased, indicating that anti-aging agents can promote the elastic deformation ability of the modified asphalt, thereby improving the resistance to rutting deformation, which is identical to the result of PG. It is noteworthy that the R of composite modified asphalt with the addition of PPA is highest, and the rutting deformation resistance is strongest. The R variation trend of modified asphalt at 3.2 kPa is consistent with 0.1 kPa. When anti-aging agents were added into the modified asphalt, its deformation recovery ability was improved to varying degrees. Moreover, with the increase in temperature, the effect of different anti-aging effects is more obvious. In addition, the R of PPA/SBS modified asphalt at three temperatures is much higher than that of nanoparticles, which will provide it with the strongest elastic deformation ability and show excellent anti-rutting performance.

The above analysis means that under the same temperature and stress conditions, adding PPA can provide SBS modified asphalt with improved elastic deformation ability and more resistance to rutting deformation. The reason why PPA can increase the recovery rate R of modified asphalt is that PPA can promote the transformation of resin in asphalt to asphaltene, which is conducive to enhancing the content of asphaltene [21]. Therefore, the high-temperature deformation resistance of modified asphalt increases.

To explore the temperature sensitivity of R to stress, the recovery rate difference index R_diff_ was introduced. The lower the R_diff_ is, the more weakly sensitive the recovery rate is to stress. The calculation results of R_diff_ are shown in Table 2. Compared with the SBS modified asphalt, the R_diff_ difference slightly increases after adding nano-ZnO, indicating that the stress sensitivity rises, as shown in Table 2. However, R_diff_ decreases slightly with the addition of nano-TiO_2_ or nano-SiO_2_. This phenomenon is because nano-ZnO mainly focuses on improving the anti-ultraviolet aging properties, while it is weaker than other nanomaterials in reducing stress sensitivity. The lowest value is PPA/SBS composite modified asphalt. R_diff_ difference at three temperatures is much lower than that of other modified asphalts, which indicates that adding PPA can effectively reduce the asphalt binder sensitivity of the recovery rate to stress, and will help to delay the rapid permanent deformation trend of asphalt caused by increased stress.

(2) J_nr_ Analysis of Non-recoverable Compliance

**Figure 2:**
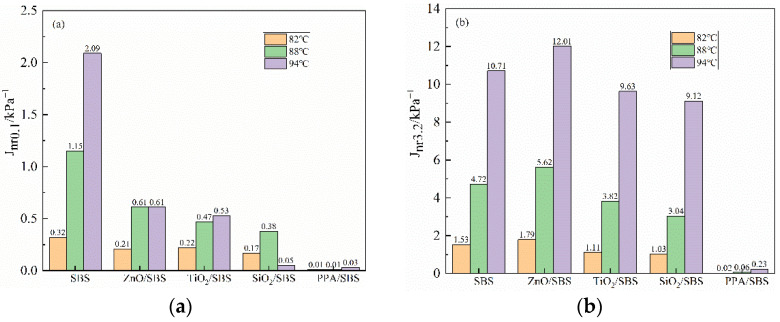
Irreversible creep compliance of asphalt at different stress levels and temperatures after RTFO aging: (**a**) J_nr0.1_; (**b**) J_nr3.2_.

Figure 2 shows the J_nr_ of the MSCR test under different stress and temperature conditions after RTFO aging of different modified asphalts. The lower the J_nr_ of the asphalt is, the less strain will be generated under stress, and the stronger the deformation resistance the asphalt will have. Compared with SBS modified asphalt, adding various anti-aging materials can reduce the irrecoverable creep compliance J_nr_ of asphalt to varying degrees under the same stress or temperature conditions, as shown in Figure 2. This means that adding different anti-aging materials will improve the ability of modified asphalt to resist rutting deformation. It is worth noting that under the same conditions, the J_nr_ of the PPA/SBS modified asphalt is two orders of magnitude lower than that of other modified asphalt. Its resistance to rutting deformation is highly excellent, which is consistent with the above analysis of the recovery rate R.

**Figure 3:**
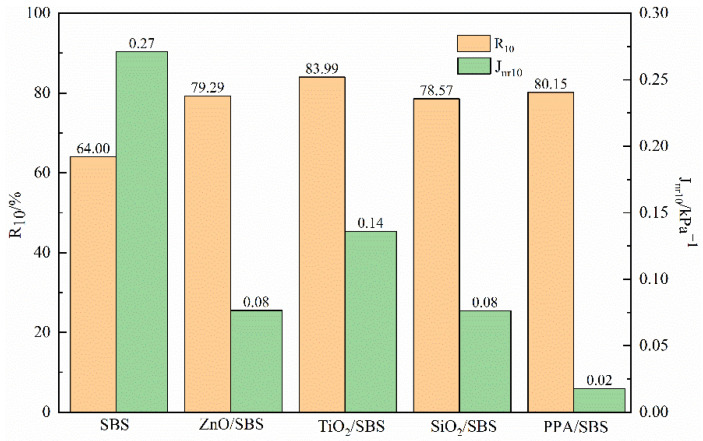
R_10_ and J_nr10_ of asphalt at 10 KPa and 70 °C after RTFO aging.

Studies have shown that the MSCR test results at higher stress correlate more with the resistance rutting test of asphalt mixtures than the standard stress required by ASTM D7405. Therefore, in order to analyze the asphalt binder’s stress dependence, the MSCR test was supplemented; it was conducted at 10 kPa and 70 °C. The results are shown in Figure 3. After increasing the stress level, there is no clear difference in the R of various asphalts added to anti-aging agents. Furthermore, compared with the SBS modified asphalt, it has a certain degree of improvement, indicating that the resistance deformation ability of the asphalt has been promoted. In addition, the non-recoverable compliance J_nr10_ index of different asphalts is quite different, and the anti-aging properties of asphalts are significantly decreased, which will greatly improve their rutting resistance. PPA/SBS composite modified asphalt has the strongest rutting resistance among different modified asphalts.

### 3.2. Effects of PAV Aging on Medium Temperature Fatigue Properties of Different High Content SBS Polymer Composite Modified Asphalts

**Figure 4:**
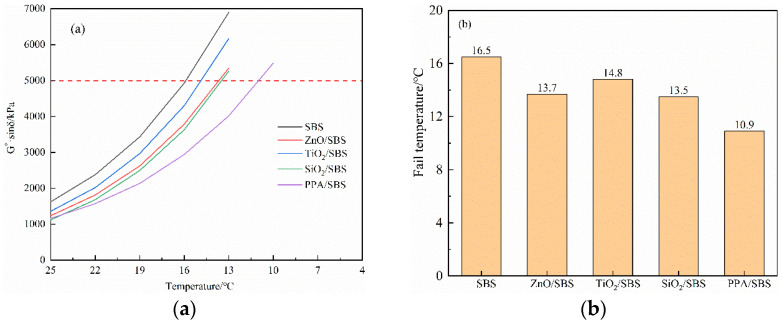
Fatigue factor (**a**) and failure temperature (**b**) of different SBS composite modified asphalts after PAV aging.

G*·sinδ is the loss shear modulus, representing the energy dissipated by the internal friction of asphalt during deformation. The higher the G*·sinδ, the greater the energy loss rate. Therefore, the lower G*·sinδ indicates better fatigue resistance. In the DSR test, the modified asphalt has fatigue failure when the fatigue factor value exceeds 5000 kPa. Figure 4 shows the fatigue factor and failure temperature of modified asphalt after PAV aging. It can be seen, from Figure 4, that the fatigue factor and the failure temperature of various anti-aging composite modified asphalts at the same temperature are significantly reduced due to the addition of anti-aging agents. Among them, PPA has the strongest fatigue resistance. Its failure temperature is as low as 10.9 °C, which is 33.9% lower than that of asphalt without any anti-aging agent, and fatigue resistance is greatly improved.

Since the fatigue factor is a linear viscoelastic index, its essence belongs to the linear viscoelastic dissipation energy. This index only uses the dissipated energy information of the specimen obtained in 10 loadings of the DSR test; however, it is hard to fully show the material state of the asphalt after tens of thousands of loading processes. So, this study introduced a DSR-based LAS. This method characterizes the fatigue life of pavement as a function of strain and uses progressively increasing pressure for periodic loading to accelerate the fatigue failure of asphalts. The test results of various modified asphalts after PAV aging are depicted in Table 3.

As shown in Table 3, the fatigue performance parameter N_f_ of composite modified asphalt at three strain levels of 2.5%, 5% and 10% increases due to the addition of anti-aging agents, meaning that their resistance fatigue performance has been improved to some extent. In addition, the N_f_ of PPA/SBS modified asphalt is clearly higher than that of other modified asphalts by two orders of magnitude, and the fatigue resistance is excellent. The reason is that, adding PPA to asphalt increases the content of asphaltenes, which is conducive to enhance its mechanical properties, and thus improves the fatigue resistance. The fatigue resistance of different modified asphalts is ranked as follows: PPA/SBS > ZnO/SBS > SiO_2_/SBS > TiO_2_/SBS > SBS modified asphalt.

### 3.3. Micromorphological Analysis of Different Polymer Modified Asphalts with a High Content of Linear SBS

**Figure 5:**
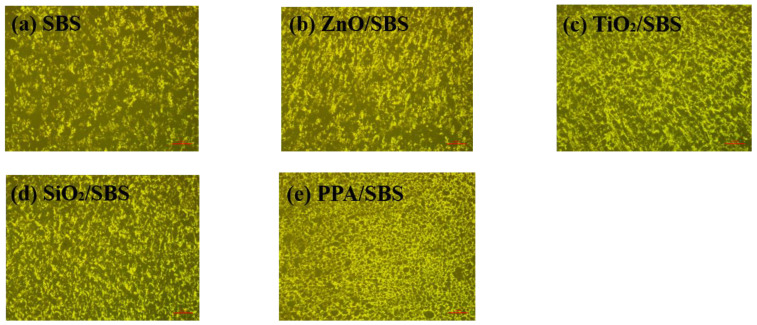
Fluorescence microscopy results of different polymer modified asphalts with a high content of linear SBS.

The micro-morphology of different modified asphalts was observed by fluorescence microscope to evaluate the influence of different anti-aging agents on the dispersion state of SBS. The results are shown in Figure 5. The fluorescent region represents the SBS phase. Different modified asphalts have apparent differences in particle size and distribution as shown in Figure 5. Figure 5a presents how the SBS dispersion in modified asphalt with a high content of linear SBS is relatively poor. In addition, there are large particle sizes and significant particle size differences, meaning that the stability of the asphalt is low. After adding nanoparticles, such as nano-ZnO, the large size particles of SBS in the composite modified asphalt decreased, indicating that these nanomaterials play a certain role in promoting dispersion after special surface modification. Among all of the modified asphalts, the PPA/SBS composite modified asphalt has the most uniform SBS distribution and the tiniest particles, forming an apparent cross-network structure, meaning that PPA reacts with SBS modified asphalt. This structure encapsulates the asphalt in the network to create a single-phase microstructure, within which SBS and asphalt are used as the continuous and dispersed phase, respectively.

In summary, SBS dispersed into finer particles and was more evenly distributed in asphalt due to the addition of anti-aging agents. Among the antioxidants, PPA has the most potent dispersing effect on SBS particles, which can significantly enhance the swelling effect of SBS. Furthermore, PPA can also improve the spatial network structure, thereby improving the storage and anti-aging performance of SBS modified asphalt.

### 3.4. Analysis of Infrared Spectroscopy of Different High Content SBS Polymer Composite Modified Asphalts

**Figure 6:**
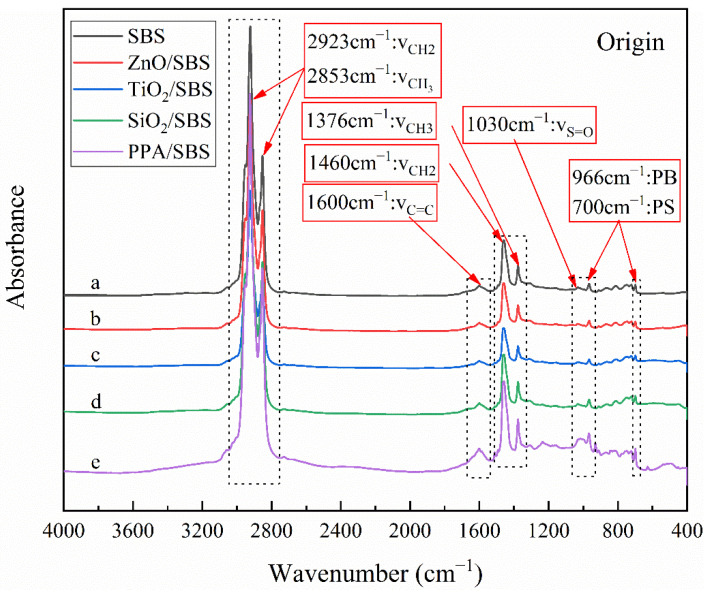
Infrared spectra of original high content SBS composite modified asphalt.

**Figure 7:**
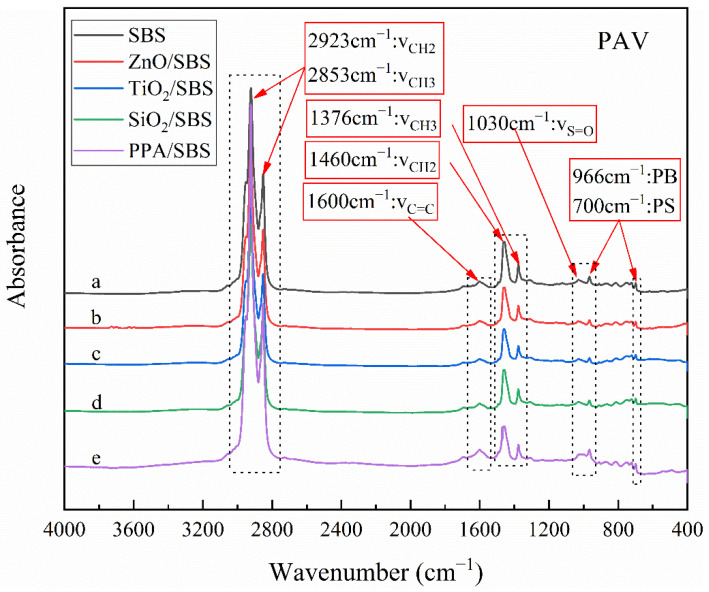
Infrared spectra of different high content SBS composite modified asphalts under a PAV aging state.

The infrared spectra of the samples were qualitatively compared at first, to research the influence of the different anti-aging agents on the chemical components of modified asphalt with a high content of linear SBS and the anti-aging effect. Considering that there is no significant difference in spectra before and after short-term aging, the samples before aging and after long-term aging are drawn here. Figure 6 and Figure 7 present the results of the infrared spectrum, and the characteristic peaks commonly used in the asphalt have also been marked in the figures.

Figure 6a shows the infrared spectrum corresponding to the original high content SBS polymer modified asphalt. In the figure, the peaks of 2923 cm^−1^ and 2853 cm^−1^ correspond to the asymmetric stretching characteristic peak of -CH_3_ and the symmetrical stretching characteristic peak of -CH_2_-, respectively. The peak at 1700 cm^−1^ belongs to the characteristic peak of the carbonyl C=O stretching vibration, and the peak at 1600 cm^−1^ belongs to the C=C stretching vibration, while two characteristic peaks near 1460 cm^−1^ and 1376 cm^−1^ originate from the asymmetric vibration of -CH_3_- and the symmetrical vibration of -CH_2_-, respectively. At the same time, there is an evident vibration characteristic peak at 1030 cm^−1^ belonging to the sulfoxide group S=O. The characteristic peaks at 966 cm^−1^ and 700 cm^−1^ originate from the bending vibration of polybutadiene PB and polystyrene PS, respectively.

Figure 6b–d correspond to the infrared spectra of nanoparticles/SBS modified asphalt. Compared with Figure 6a, it is easy to notice that the infrared spectrum basically overlaps between high content SBS modified asphalt with and without nano materials. There are no new characteristic peaks generated in their spectra, and the intensities of the existing characteristic peaks do not change significantly, which indicates that the addition of nanoparticles does not generate new substances in modified asphalt, and they are simply physically blended with the asphalt. Therefore, the enhancement of the anti-aging performance of modified asphalt after adding nanoparticles may be due to the synergistic effect of surface effect, volume effect, quantum size effect, and macroscopic quantum tunneling effect of nanoparticles.

Figure 6e depicts that the mid-infrared absorption peak of the PPA/SBS composite modified asphalt has an -OH vibration at 3900~3400 cm^−1^, which is the strong vibration region of PPA. In addition, most of the absorption peaks are in agreement with SBS modified asphalt, indicating a physical modification phenomenon. The intensity of the peak at 1100~1000 cm^−1^ corresponded to an -OH change, and the relative intensity of the two peaks changed with the addition of PPA, which indicated the existence of physical modification and partial chemical modification. It is mainly the out-of-plane bending vibration absorption peak of unsaturated C-H (=C-H) at less than 1000 cm^−1^ and the S=O vibration in the sulfoxide group near 1030 cm^−1^. There are relatively great and weak changes in the intensity of these regions, and their peak types are unchanged. However, the contents are different, indicating that the addition of PPA forms chemical cross-links. Therefore, after adding PPA to SBS modified asphalt, physical and chemical modifications are caused. The IR spectrum of asphalt show slight absorption at the strong absorption of SBS and PPA due to physical modification; it is a pity that the change is insignificant. The chemical modification is reflected in that the relative absorption intensity of the peak changes after adding PPA.

Figure 7 shows the infrared spectra of different composite modified asphalts under a PAV aging state. Compared with the original modified asphalt with a high content linear SBS, the absorption peak intensity of C=O (1700 cm^−1^) and S=O (1030 cm^261^) increases, which corresponds to the hardening of the asphalt phase. It is also reflected in the reduction of the polystyrene (700 cm^−1^) and polybutadiene peaks (966 cm^−1^) due to the degradation of the polymer phase. By comparison, it is found that when the modified asphalt was aged by PAV, the SBS is seriously degraded, leading to insufficient anti-aging ability.

At the same time, comparing different nanoparticle composite modified asphalts, it can be found that although the area of the polybutadiene peak (966 cm^−1^) decreased after aging, it was slightly higher than that of modified asphalt without nanoparticles, indicating that the SBS degradation degree becomes slow due to the addition of nanoparticles. In addition, the peak area of different asphalts decreased to a similar extent, indicating that nanoparticles, such as nano-ZnO, have no obvious difference in anti-aging property. The degree of reduction in the peak area of different pitches is relatively close, indicating that the anti-aging ability is not much different. All of them can slightly enhance the anti-aging ability, but the effect is insignificant. It should be pointed out that their polystyrene characteristic peak (700 cm^−1^) does not change significantly. This is mainly because the first hydrogen atom (alpha hydrogen) connected to the carbon–carbon double bond functional group on the polybutadiene segment is chemically active and easily attacked by oxygen to generate oxides, which results in the bond breaking. In contrast, polystyrene is relatively stable in thermal-oxidative aging.

The previous research proved that the anti-aging performance of low content SBS (<5.0 wt%) modified asphalt is effectively improved by nanoparticles. This is because nanoparticles with a high surface energy and large active specific surface area have a strong bonding ability to both asphalt and polymer particles. The addition of nanoparticles can form an oriented film between the polymer and asphalt, thus reducing the interfacial tension between the polymer and asphalt and improving the dispersion of polymer particles, which is consistent with the results of the fluorescence microscopy test in this paper [15]. However, for high content SBS modified asphalt with a strong network cross-link structure, SBS as a continuous phase, and asphalt as a dispersed phase, the movement of SBS molecules is blocked, and the adsorption effect of the nano materials is weakened. Therefore, nanoparticles slightly improve the performance of high content SBS modified asphalt. Interestingly, compared with the original modified asphalt, after PAV aging, the infrared spectrum difference of SBS modified asphalt with nano materials is reduced, which indicates that nano materials have no significant impact on aging resistance. This result is consistent with the fatigue resistance of modified asphalt with or without nano materials after PAV aging, as shown in Table 3.

By comparing the spectra of PPA/SBS composite modified asphalt before and after aging, it can be seen that the infrared spectrum of the aged asphalt almost coincides with the original one. Its carbonyl characteristic peaks, polystyrene peaks and polybutadiene peaks hardly change, and its macroscopic performance is far superior to other modified asphalts. Combined with the fluorescence microscope results and infrared results, it is not difficult to find that the mechanism of PPA within high content SBS modified asphalt is that PPA reacts with the S=O in asphaltene, which enhances the cross-linking effect of the SBS in the asphalt.

**Figure 8:**
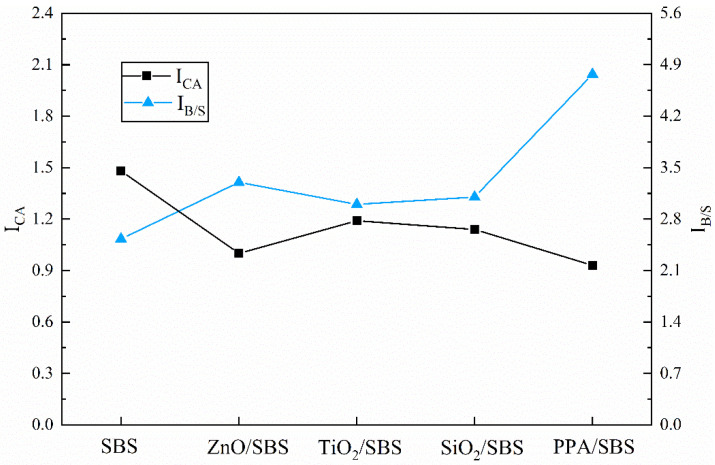
Quantitative analysis results of infrared spectra of different high content SBS composite modified asphalts under PAV aging state.

To further evaluate the influence of anti-aging agents on the anti-aging performance of modified asphalt, the aging behavior of samples were semi-quantitatively analyzed by calculating the carbonyl index and the I_B/S_ index. The results are depicted in Figure 8. During the aging process, asphalt undergoes an oxygen absorption reaction, producing oxygen-containing components such as aldehydes and ketones, which manifest the -OH (at 1700 cm^−1^) content increases. It is also accompanied by the degradation of SBS, reflected in the reduction of the polystyrene and polybutadiene peaks However, the decline of the peak at 700 cm^−1^ is much lower than that of 966 cm^−1^ due to the lower sensitivity of polystyrene to aging. Therefore, the smaller the carbonyl index and the larger the I_B/S_ index, the lower the oxidation degree of the asphalt and the higher the integrity of the SBS. According to the test results in Figure 8, the addition of nanomaterials, such as nano-ZnO, can appropriately reduce the carbonyl index and increase the I_B/S_ index, and the anti-aging performance of the asphalt is slightly improved. The carbonyl index of the PPA/SBS modified asphalt is the lowest, and the I_B/S_ index is clearly higher than that of others. SBS content is the highest after aging, and its anti-aging ability is exceptionally outstanding. This is because adding PPA will produce a chemical cross-linking reaction, which can effectively prevent the oxidation of the asphalt and the decomposition of the SBS.

The above qualitative analysis of the infrared spectra of modified asphalts suggests that there is only a simple physical blending of nanoparticles and SBS modified asphalt, while PPA can play a role in physical and chemical modification at the same time. The addition of PPA can effectively reduce the influence of thermal-oxidative aging on asphalt properties and significantly improve the anti-aging properties.

## 4. Conclusions

In this paper, from the perspective of rheology and microchemical analysis, the influence of nano-ZnO, nano-TiO_2_ and nano-SiO_2_ and PPA on the anti-aging properties of modified asphalt with a high content of linear SBS were evaluated, and the modification mechanism was explained. The major conclusions were drawn as follows:

(1) No matter whether nanoparticles are added or not, the PG temperature of high content SBS modified asphalt after short-term aging is 82 °C, which implies that the nanoparticles have no significant effect on high-temperature rutting resistance. After PAV aging, the anti-fatigue factor is in the same order of magnitude. However, PPA significantly improved PG temperature (124 °C) and high-temperature rutting resistance. Compared with other SBS modified asphalts, the fatigue property parameter N_f_ was improved by two orders of magnitude. In other words, nano materials cannot significantly improve the anti-aging performance of high content SBS modified asphalt. On the contrary, PPA has excellent anti-aging performance.

(2) The infrared spectrum of high content SBS modified asphalt with and without nanoparticles basically overlaps after aging, indicating that nanoparticles have hardly any anti-aging performance. The reason is that the nanoparticles and high content SBS modified asphalt are simply mixing physically; the movement of the SBS molecules is blocked by the strong network cross-linking structure, which leads to a reduction of the adsorption effect of the nanoparticles.

(3) By comparing the spectra of the PPA/SBS composite modified asphalt before and after aging, it can be found that the carbonyl characteristic peaks, polystyrene peaks and polybutadiene peaks have little change, and its macroscopic mechanical performance is far superior to other modified asphalts, meaning that PPA has outstanding anti-aging performance. This is because PPA reacts with the S=O in asphaltene, which enhances the cross-linking effect of the SBS in asphalt.

## Figures and Tables

**Table 1:** Rheological properties of all asphalt samples in RFTO aging state.

Samples	δ/°	G*/Kpa	G*/Sin δ/Kpa	Failure Temperature/°C
SBS	63.10	2.74	3.07	86.1
ZnO/SBS	64.31	2.39	2.65	84.4
TiO_2_/SBS	56.80	2.32	2.78	85.6
SiO_2_/SBS	57.20	2.41	2.87	85.9
PPA/SBS	40.20	16.05	24.86	125.7

**Table 2:** Recovery rate difference R_diff_ of various modified asphalts at different temperatures.

Samples	R_diff_ (%)		
	82 °C	88 °C	94 °C
SBS	44.1	72.49	94.04
ZnO/SBS	49.87	83.76	97.70
TiO_2_/SBS	35.70	68.60	92.62
SiO_2_/SBS	37.31	66.37	90.30
PPA/SBS	10.10	21.93	41.73

**Table 3:** LAS test results of different asphalts after long-term aging.

Samples	A	B	α	2.5%N_f_	5%N_f_	10%N_f_
SBS	8.341 × 10^6^	3.648	1.824	294,850	23,522	1877
ZnO/SBS	2.524 × 10^7^	3.775	1.888	793,882	57,976	4234
TiO_2_/SBS	9.978 × 10^6^	3.588	1.794	372,679	30,997	2578
SiO_2_/SBS	1.385 × 10^7^	3.483	1.741	569,319	50,919	4554
PPA/SBS	1.252 × 10^10^	5.423	2.712	87,004,865	2,027,422	47,244

## Data Availability

The data that supports the findings of this study are available within the article.
